# Molecular docking analysis of flavonoids with AChE and BACE-1

**DOI:** 10.6026/973206300200103

**Published:** 2024-02-29

**Authors:** Sittarthan Viswanathan, Thennavan Arumugam, Kavimani Subramanian, Rengaraj Sivaraj, Vimalavathini Ramesh, A. Hannah Rachel Vasanthi

**Affiliations:** 1 Department of Pharmacology, Mother Theresa Post Graduate & Research Institute of Health Sciences (Government of Puducherry Institution), Puducherry - 605006, India; 2 Department of Pharmacology, Central Animal House, JIPMER, Puducherry - 605006; 3 Department of Pharmacology, Aarupadai Veedu Medical College & Hospital, Puducherry 607402; 4 Department of Biotechnology, Pondicherry University - 605014

**Keywords:** Alzheimer's, disease, Flavonoids, BACE-1, AChE, OSIRIS

## Abstract

Flavonoids are promising therapeutics for the treatment of Alzheimer's disease (AD). Therefore, it is of interest to study the anti-AD potential of 35 flavonoids towards the inhibition of AchE and BACE-1. Hence, the physicochemical, pharmacokinetic parameters, toxicity risk and drug-likeliness of the selected 35 flavonoids were computed. Further, the molecular docking analysis of flavonoids with AChE and BACE-1 were completed. A binding energy of -10.42 kcal/mol Epicatechin gallate, -10.16 kcal/mol sterubin and -10.11 kcal/mol Fisetin was observed with AchE as potential inhibitors. Similarly, Biochainin-A -9.81kcal/mol, Sterubin -8.96 kcal/mol and Epicatechin gallate -7.4 7 kcal/mol showed with BACE-1. Thus, these flavonoids are potential leads for structure-based design of effective anti-Alzheimer's agents.

## Background:

Alzheimer's disease (AD) is a chronic, progressive, irreversible neurodegenerative disorder. It is characterized by immense loss of functional deficits, and slowly destroys memory, thinking skills and eventually the ability to behaviour. The two major hallmarks of AD's is amyloid-(β) peptide-containing extracellular amyloid plaques and hyper-phosphorylated tau (p-tau) protein-containing intracellular neurofibrillary tangles (NFT) are formed in the brain [1]. The United States Food and Drug Administration (FDA) have thus far approved two categories of medications for the treatment of AD: Acetylcholinesterase inhibitors and N-methyl-D-aspartate (NMDA) receptor antagonists [2]. However, these drug classes can only provide limited and temporary relief from symptoms, in addition to causing undesirable side effects [3]. Additionally, the existing treatments for AD are only partially effective and cannot halt, reverse, or prevent the progression of the disease [4]. The neurotransmitter acetylcholine (Ach) plays an important role in learning and memory in the hippocampus. Under normal physiological conditions, Ach is hydrolysis by cholinesterase's are a common class of serine hydrolases that break down choline esters. The two most well-known types are acetylcholinesterase (AChE) and butyrylcholinesterase (BChE). Acetylcholine (ACh) is hydrolysed by AChE into choline and acetate, eliminating the neurotransmitter's action at cholinergic synapses; BChE performs some of the same functions, but its function in the brain is still unknown. The "Cholinergic hypothesis of AD" states that AD causes severe damage to cholinergic neurons in the basal forebrain, followed by neuronal loss and a decrease in the ACh synthesis and degeneration [5].

Amyloid beta (Aβ) peptides are generated through proteolytic cleavage of amyloid precursor proteins (APP) by beta-secretase (BACE-1) in a healthy brain. Usually, the proteolytic processing of APP is regulated by alpha-secretase; however, when this is suppressed, beta- and gamma-secretases take over and produces neurotoxic Aβ40/42 peptides. The sequential proteolysis of APP into Aβ40/42 is mediated by: (i) BACE-1, which cleaves APP extracellularly at Asp671 to produce a 99 amino acid beta-carboxyl terminal fragment (c99), and (ii) gamma-secretase, which cleaves c99 to release βA peptides into the extracellular space. These βA peptides then accumulate to form toxic senile plaques outside the cells [6]. In the fields of nutrition and medicine, natural herbs are crucial. The flavonoids, phenolics, alkaloids, and tannins are the chemical components of herbs that are most important. Flavonoids are polyphenolic substances that are widely found in natural herbs and are an essential component of a regular human diet. It consists of two aromatic rings including benzopyran and benzene [7, 8,9]. Flavonoids align with the characteristics of NDDs by suppressing lipid peroxidation, inhibiting inflammatory mediators, modulating gene expression, and activating antioxidant enzymes. As a result, flavonoids support the maintenance of neuron's endogenous antioxidant status, safeguarding them against neurodegeneration [10,11]. Accordingly, flavonoids are subdivided into the following subgroups; flavonols (e.g. quercetin), flavones (e.g. apigenin), isoflavones (e.g. genistein), flavanones (e.g. hesperetin), flavanols (e.g. catechin), anthocyanidins (e.g. pelargonidin), 3-hydroxy derivatives of flavan (e.g. catechin). Numerous studies have revealed that flavonoids have a wide range of pharmacological properties, involving antioxidant, anti-inflammatory, hepatoprotective, antiangiogenic, anti-diabetic, cardioprotective, neuroprotective, and anti-Alzheimer's characteristics [12, 13]. Moreover, it has been known that AChE and BACE-1 are linked to amyloid plaques and cholinergic dysfunction, which seems to encourage the development of amyloid Aβ fibrils. The most effective approach to suppress the development of Aβ42 is anti-amyloid treatment. BACE-1 inhibition is thought to be one of the most successful ways to treat AD according to the amyloid hypothesis. BACE-1 inhibitors are efficient in combating new Aβ plaques but inefficient against growth of existing plaques, suggesting early treatment with the aim of preventing initial plaque formation. Thus, inhibition of both enzymes is a highly desirable feature of AD therapy [14]. Therefore, it is of interest to investigate the potential interaction between natural phyto-constituents with AchE and BACE-1 protein linked with AD.

## Methodology:

## Software and Hardware:

We used online databank such as PubChem [15] and Protein Data Bank [16], Online tools such as Swiss ADMET [17], [18] OSIRIS Data Warrior 4.7.3. Using software like, AutoDockTools-1.5.6 else using protein visualizer Bio-Discovery studio.

## Retrieval of target enzyme structures:

Protein Data Bank was used for retrieving the structure of the following enzymes involved in the pathogenies of AD's of Homo sapiens origin, which are recognized as targets of AChE (4EY7) & BACE-1 (5HDZ). All water molecules and hetero atom removed.

## Retrieval of ligand:

The structures of 36 Compounds were retrieved from the PubChem database. These structures were used for docking studies. The selected 3D structure of the ligands was retrieved from PubChem compound database in SDF format followed by conversion in the PDB format and optimization using Bio-Discovery Studio. Structure, Compound, Molecular formula, and PubChem ID of Flavonoids present in the study shown in Table 1.

## Prominent active site prediction:

Prior to docking analysis, prominent active site prediction of AChE & BACE-1 were carried out by PDB Sum database https://www.ebi.ac.uk/thornton-srv/databases/cgi-bin/pdbsum/GetPage.pl?pdbcode=index.html [19].

## ADME properties:

In order to predict the ADME properties of the selected flavonoid, Swiss ADME web tool was used. It is a free web tool to compute pharmacokinetics, ADME properties, drug-likeliness, and medicinal chemistry friendliness of small molecules. The import tool on the input zone of the Swiss ADME submission page was used to retrieve the compound structure from databases, converted into SMILES format databases, converted into SMILES format and then calculations were run. In some cases, the SMILE format of the compound was copied from the PubChem database and directly pasted before running. When results were loaded, they were saved as a CSV file.

## Toxicity risks assessment and drug likeliness:

To assess the toxicity risks of the selected flavonoid, their SMILES were retrieved from PubChem database and illustrated in the OSIRIS Property Explorer open source program which computes toxicity risks and drug-relevant properties of compounds and provides results as safe, mild and moderate coded features.

## Molecular docking:

## Ligand Preparation:

Compound from different groups of the flavonoid family such as flavonones, flavonols, flavone, isoflavone, anthocyanins, flavanols, flavanonol and chalcones selected to test for their inhibitory capabilities among the selected protein. The 2-dimensional structures (2D) of 35 flavonoids were retrieved from the NCBI PubChem database in .sdf format. Whereas, the 2D structure of the ligands was prepared and converted into PDB file using Bio-Discovery Studios.

## Molecular docking:

Molecular docking was performed using Autodock 1.5.6 software, based on Lamarckian Genetic Algorithm, which combines energy evaluation through grids of affinity potential to find the suitable binding position for a ligand on a given protein. Polar hydrogen atoms were added to protein targets and kollman united atomic charges were computed. The grid dimensions were 60 Å X 60 Å X 60 Å with points separated by 0.375 Å. The grid box was then allocated properly in the target to include the active residue in the center. For all ligands, random starting positions, random orientations and torsions were used. The Docking parameters Number of Genetic Algorithm (GA) runs: 25, Population size: 150, Maximum number of evaluations: 2,500,000, Maximum number of generations: 27,000 were used for this study. The structure with the lowest binding free energy and the most cluster members was chosen for the optimum docking conformation. Finally, results were visualized using Visualizer Bio-Discovery Studio [20, 21,22,23, 24].

## Results & Discussion:

**Figure 1:**
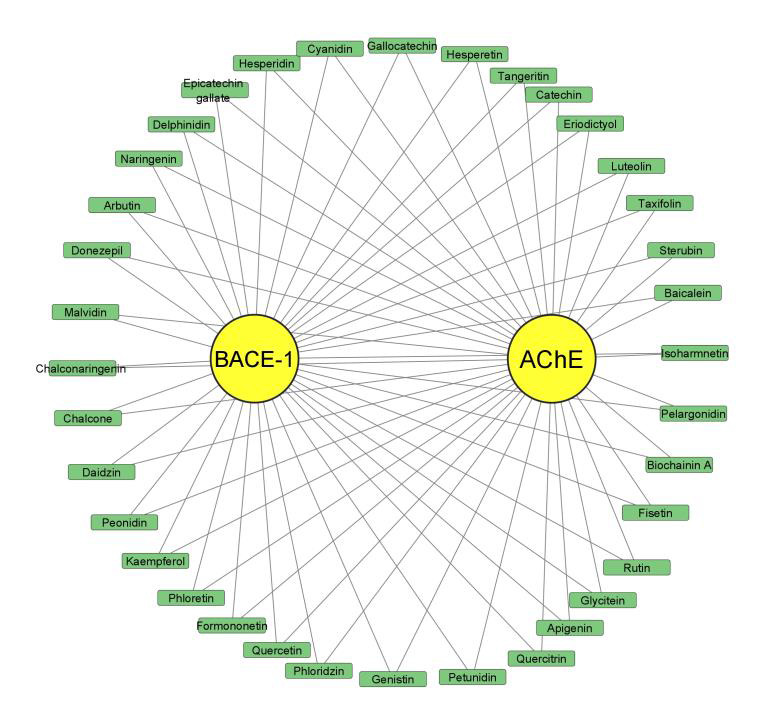
35 Flavonoids were docked with acetylcholinesterase and β-secretase protein

The main objective of this study was to explore the potential of flavonoids as a treatment for Alzheimer's disease by examining their interactions with essential proteins involved in the disease. Specifically, this study focused on docking 35 flavonoids with acetylcholinesterase and β-secretase, as shown in Figure 1. The pharmacokinetic properties of natural compounds to be considered drug candidates were based on Lipinski's Rule of Five (RO5). The Lipinski rule of five was applied to the 35 selected flavonoids using SwissADME software. The results, including Lipinski (RO5) and physicochemical properties of the docked compounds, are presented in Table 2 and Table 3, respectively. Molecules that violate more than one of these rules may cause bioavailability problems. The entire set of compounds well followed the RO5 except 7 of the compounds, out of which four compounds (Hesperidin, Procyanidin, Quercetin and Rutin) violated more than one of these rules and three compounds violated only a single rule (Phloridzin, Dephilidin and Genistein) that created the Lipinski's rule violation by having, and that can make a problem in oral bioavailability. Table 4 shows the toxicity profiles of the compounds obtained using the OSIRIS Property Explorer. This includes assessment of the potential risks of mutagenicity, tumorigenicity, irritancy, and reproductive toxicity. Among these compounds Apigenin, Kaempferol, Isoharmnetin, Fisetin and Naringenin have high risk of mutagenicity. Arbutin, Glycitein, Daidzin have a high risk of reproductive toxicity, whereas phloridzin has a mild risk of reproductive toxicity. Tangeritin and Quercetin have a high risk of mutagenic and tumorigenic effects, respectively. Genistin exhibits both tumorigenic and reproductive toxicities. However, these compounds are at high risk and do not possess good drug profiles.

**Figure 2:**
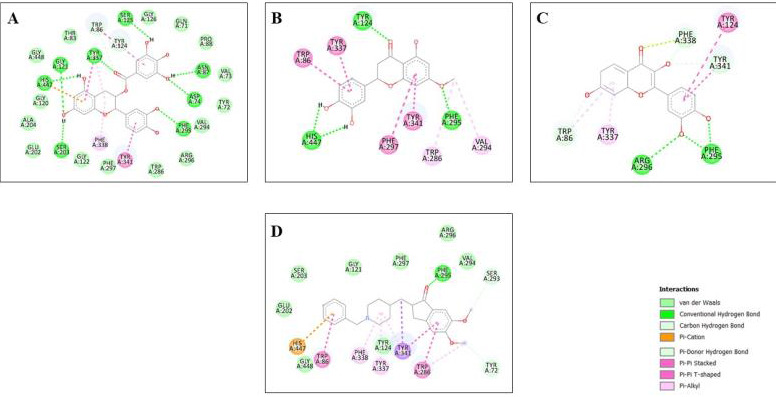
2-D interaction diagram of top 3 ranked screened flavonoids (A) Epicatechin gallate, (B) Sterubin (C) Fisetin interacted with AChE (4EY7) and (D) Donepezil respectively.

**Figure 3:**
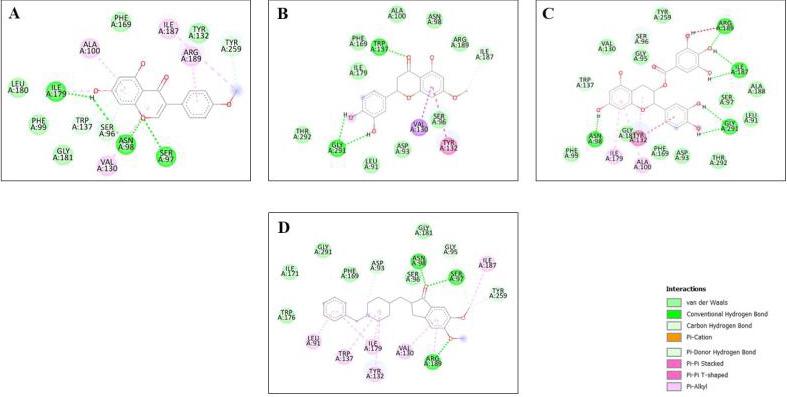
2-D interaction diagram of top 3 ranked screened flavonoids (A) Fisetin, (B) Sterubin and (C) Epicatechin gallate interacted with BACE-1 (5HDZ) and (D) Donepezil respectively.

Molecular docking was employed using Auto-dock 1.5.6 in order to predict the interactions of the protein with its ligands. The binding mode competency of AChE, BACE-1, and the flavonoids were investigated via molecular docking. The flavonoids chosen were docked with 25 Run and compared with the reference standard, donepezil. The docking energies of the selected flavonoids indicated high binding affinities to the target receptor, as shown in Table 5. Among the docked compounds for both proteins, the top 3 ligands for AChE targets (Epicatechin galate -10.42 kcal/mol, Sterubin -10.16 kcal/mol and Fisetin -10.11 kcal/mol) and BACE-1 (Fisetin -9.81 kcal/mol, Sterubin -8.96 kcal/mol, Epicatechin gallate -7.47 kcal/mol) were compared with Donepezil as shown in 2D structures (Figure 2 and Figure 3) which depict non-covalent interactions such as van der Waals, columbic contacts, u-u interactions, and hydrogen interactions. These compounds do not have toxicity and possessed large binding energy towards the target being studied. Certain critical amino acids in the ligand-binding domains of human AchE and BACE-1 have also been identified. The major non-covalent interactions between the examined ligands and the AchE and BACE-1 ligand binding domains were explored. These amino acids have been involved in ligand interactions with AchE and BACE-1, as well as in the inhibition of the ligand-binding domains of AchE and BACE-1. In patients with Alzheimer's disease, plaques block the connections of neurons, causing messages to be delayed or lost. By reducing the breakdown of acetylcholine to the choline moiety, AChE inhibition can improve signalling [25]. Beta-secretase is known to cause the cleavage of beta-amyloid protein into beta-amyloid plaques, which are a characteristic of Alzheimer's and dementia. By suppressing beta-secretase activity, it is possible to prevent the transformation of amyloid precursor protein into insoluble beta-amyloid [26, 27,28,29].

## Conclusion:

Data shows that the flavonoids epicatechin gallalate, sterubin and biochanin A have high binding with targets linked with Alzheimer's disease for further considerations *in vitro* and *in vivo*.

## Disclosure statement:

The authors declare no conflicts of interests.

## Ethical approval:

This article does not contain any human participant and animal work.

## Author contributions:

All author contributed equally.

## Funding:

None declared or Self-funding

## Data availability statement:

All dataset supporting this article is available within the article and its supplementary files.

## Figures and Tables

**Table 1:** Compound, Molecular formula, and PubChem ID of Flavonoids present in the study.

**Sl. NO**	**Compound**	**Molecular formula**	**PubChem ID**
1	Petunidin	C16H13O7	441774
2	Peonidin	C16H13O6	441773
3	Pelargonidin	C15H11O5	440832
4	Malvidin	C17H15O7	159287
5	Delphinidin	C15H11ClO7	68245
6	Cyanidin	C15H11O6	128861
7	Tangeritin	C20H20O7	68077
8	Luteolin	C15H10O6	5280445
9	Baicalein	C15H10O5	5281605
10	Apigenin	C15H10O5	5280443
11	Phloridzin	C21H24O10	6072
12	Phloretin	C15H14O5	4788
13	Chalcone	C15H12O	637760
14	Chalconaringenin	C15H12O5	5280960
15	Arbutin	C12H16O7	440936
16	Epicatechin gallate	C22H18O11	65064
17	Gallocatechin	C15H14O7	65084
18	Catechin	C15H14O6	73160
19	Taxifolin	C15H12O7	439533
20	Rutin	C27H30O16	5280805
21	Quercitrin	C21H20O11	5280459
22	Quercetin	C15H10O7	5280343
23	Kaempferol	C15H10O6	5280863
24	Isoharmnetin	C16H12O7	5281654
25	Fisetin	C15H10O6	5281614
26	Naringenin	C15H12O5	932
27	Hesperidin	C28H34O15	10621
28	Hesperetin	C16H14O6	72281
29	Eriodictyol	C15H12O6	440735
30	Sterubin	C16H14O6	4872981
31	Glycitein	C16H12O5	5317750
32	Genistin	C21H20O10	5281377
33	Formononetin	C16H12O4	5280378
34	Daidzin	C15H10O4	5281708
35	Biochainin A	C15H10O4	5280373
36	Donepezil	C24H29NO3	3152

**Table 2:** Physicochemical properties of flavonoids

**Sl. No**	**Ligand**	**Molecular weight**	**No of rotatable bonds**	**H bond donor**	**H bond acceptor**	**clog P**	**Solubility log S**	**TPSA**
1	Petunidin	317.27	2	5	7	1.57	-3.27	110.38
2	Peonidin	301.27	2	4	6	1.91	-3.57	90.15
3	Pelargonidin	271.25	1	4	5	1.98	-3.55	80.92
4	Malvidin	331.3	3	4	7	1.84	-3.59	99.38
5	Delphinidin	338.7	1	6	7	1.29	-2.96	121.38
6	Cyanidin	287.25	1	5	6	1.64	-3.25	101.15
7	Tangeritin	372.37	6	0	7	3.02	-3.83	72.45
8	Luteolin	286.24	1	4	6	1.99	-2.56	107.22
9	Baicalein	270.24	1	3	5	2.34	-2.86	86.99
10	Apigenin	270.24	1	3	5	2.34	-2.86	86.99
11	Phloridzin	436.41	7	7	10	0.06	-2.41	177.14
12	Phloretin	274.27	4	4	5	2.04	-2.52	97.99
13	Chalcone	208.26	3	0	1	3.3	-3.84	17.07
14	Chalconaringenin	272.26	3	4	5	1.92	-2.66	97.99
15	Arbutin	272.25	3	5	7	-1.02	-0.91	119.61
16	Epicatechin gallate	458.37	4	8	11	2.05	-2.16	197.37
17	Gallocatechin	306.27	1	6	7	1.16	-1.47	130.61
18	Catechin	290.27	1	5	6	1.51	-1.76	110.38
19	Taxifolin	304.25	1	5	7	0.96	-1.94	127.45
20	Rutin	610.52	6	10	16	-1.26	-2.4	265.52
21	Quercitrin	448.38	3	7	11	0.58	-2.7	186.37
22	Quercetin	302.24	1	5	7	1.49	-2.49	127.45
23	Kaempferol	286.24	1	4	6	1.84	-2.79	107.22
24	Isoharmnetin	316.26	2	4	7	1.77	-2.8	116.45
25	Fisetin	286.24	1	4	6	1.84	-2.79	107.22
26	Naringenin	272.26	1	3	5	2.16	-2.64	86.99
27	Hesperidin	610.56	7	8	15	-0.81	-2.75	234.29
28	Hesperetin	302.28	2	3	6	2.09	-2.66	96.22
29	Eriodictyol	288.25	1	4	6	1.81	-2.34	107.22
30	Sterubin	302.28	2	3	6	2.09	-2.66	96.22
31	Glycitein	284.27	2	2	5	1.9	-3.04	75.99
32	Genistin	432.38	4	6	10	-0.36	-2.61	166.14
33	Formononetin	268.27	2	1	4	2.25	-3.34	55.76
34	Daidzin	254.24	1	2	4	1.97	-3.02	66.76
35	Biochainin A	284.27	2	2	5	1.9	-3.04	75.99
36	Donezepil	379.5	6	0	4	4.21	-4.35	38.77

**Table 3:** Predicted ADME Properties of flavonoids

**Sl.No**	**Ligand**	**HIA**	**BBB Permeate**	**P-gp Substrate**	**CYP1A2 inhibitor**	**CYP2C19 inhibitor**	**CYP2C9 inhibitor**	**CYP2D6 inhibitor**	**CYP3A4 inhibitor**	**Log Kp (Skin permeation cm/s)**
1	Petunidin	High	No	Yes	Yes	No	No	No	No	-6.88 cm/s
2	Peonidin	High	No	Yes	Yes	No	No	No	No	-6.53 cm/s
3	Pelargonidin	High	No	Yes	Yes	No	No	Yes	No	-6.33 cm/s
4	Malvidin	High	No	Yes	Yes	No	No	No	No	-6.73 cm/s
5	Delphinidin	High	No	Yes	No	No	No	No	No	-7.50 cm/s
6	Cyanidin	High	No	Yes	Yes	No	No	No	No	-7.51 cm/s
7	Tangeritin	High	Yes	No	No	No	Yes	No	Yes	-6.41 cm/s
8	Luteolin	High	No	No	Yes	No	No	Yes	Yes	-6.25 cm/s
9	Baicalein	High	No	No	Yes	No	No	Yes	Yes	-5.70 cm/s
10	Apigenin	High	No	No	Yes	No	No	Yes	Yes	-5.80 cm/s
11	Phloridzin	Low	No	Yes	No	No	No	No	No	-8.58 cm/s
12	Phloretin	High	No	No	Yes	No	Yes	No	Yes	-6.11 cm/s
13	Chalcone	High	Yes	No	No	Yes	No	No	No	-5.38 cm/s
14	Chalconaringenin	High	No	No	Yes	No	Yes	NO	Yes	-5.96 cm/s
15	Arbutin	High	No	No	No	No	No	No	No	-8.92 cm/s
16	Epicatechin gallate	Low	No	No	No	No	No	No	No	-8.27 cm/s
17	Gallocatechin	High	No	No	No	No	No	No	No	-8.17 cm/s
18	Catechin	High	No	Yes	No	No	No	No	No	-7.82 cm/s
19	Taxifolin	High	No	No	No	No	No	No	No	-7.48 cm/s
20	Rutin	Low	No	Yes	No	No	No	No	No	-10.2 cm/s
21	Quercitrin	Low	No	No	No	No	No	No	No	-8.42 cm/s
22	Quercetin	High	No	No	Yes	No	No	Yes	Yes	-7.05 cm/s
23	Kaempferol	High	No	No	Yes	No	No	Yes	Yes	-6.70 cm/s
24	Isoharmnetin	High	No	No	Yes	No	No	Yes	Yes	-6.90 cm/s
25	Fisetin	High	No	No	Yes	No	No	Yes	Yes	-6.65 cm/s
26	Naringenin	High	No	No	Yes	No	Yes	No	Yes	-5.96 cm/s
27	Hesperidin	Low	No	Yes	No	No	No	No	No	-10.12 cm/s
28	Hesperetin	High	No	Yes	Yes	No	No	No	Yes	-6.30 cm/s
29	Eriodictyol	High	No	Yes	No	No	No	No	Yes	-6.62 cm/s
30	Sterubin	High	No	Yes	Yes	No	No	No	Yes	-6.48 cm/s
31	Glycitein	High	No	No	Yes	No	No	Yes	Yes	-6.30 cm/s
32	Genistin	Low	No	No	No	No	No	No	No	-8.33 cm/s
33	Formononetin	High	Yes	No	Yes	No	No	Yes	Yes	-5.95 cm/s
34	Daidzin	High	Yes	No	Yes	No	No	Yes	Yes	-6.10 cm/s
35	Biochainin A	High	No	No	Yes	No	No	Yes	Yes	-5.91 cm/s
36	Donepezil	High	Yes	Yes	No	No	No	Yes	Yes	-5.58 cm/s

**Table 4:** Toxicity risks predicted by OSIRIS Property Explorer

**Sl.No**	**Ligand**	**Mutagenicity**	**Tumorigenicity**	**Skin Irritation**	**Reproductive effective**
1	Petunidin	Safe	safe	Safe	safe
2	Peonidin	Safe	safe	Safe	safe
3	Pelargonidin	Safe	safe	Safe	safe
4	Malvidin	Safe	safe	Safe	safe
5	Delphinidin	Safe	safe	Safe	safe
6	Cyanidin	Safe	safe	Safe	safe
7	Tangeritin	Mutagenic	Tumorigenic	Safe	safe
8	Luteolin	Safe	safe	Safe	safe
9	Baicalein	Safe	safe	Safe	safe
10	Apigenin	Mutagenic	safe	Safe	safe
11	Phloridzin	Safe	safe	Safe	Mild
12	Phloretin	Safe	safe	Safe	safe
13	Chalcone	Safe	safe	Safe	safe
14	Chalconaringenin	Safe	safe	Safe	safe
15	Arbutin	Safe	safe	Safe	Yes
16	Epicatechin gallate	Safe	safe	Safe	safe
17	Gallocatechin	Safe	safe	Safe	safe
18	Catechin	Safe	safe	Safe	safe
19	Taxifolin	Safe	safe	Safe	safe
20	Rutin	Safe	safe	Safe	safe
21	Quercitrin	Safe	safe	Safe	safe
22	Quercetin	Mutagenic	Yes	Safe	safe
23	Kaempferol	Mutagenic	safe	Safe	safe
24	Isoharmnetin	Mutagenic	safe	Safe	safe
25	Fisetin	Safe	safe	Safe	safe
26	Naringenin	Mutagenic	safe	Safe	safe
27	Hesperidin	Safe	safe	Safe	safe
28	Hesperetin	Safe	safe	Safe	safe
29	Eriodictyol	Safe	safe	Safe	safe
30	Sterubin	Safe	safe	Safe	safe
31	Glycitein	Safe	safe	Safe	Yes
32	Genistin	Safe	Yes	Safe	Yes
33	Formononetin	Safe	safe	Safe	safe
34	Daidzin	Safe	safe	Safe	Yes
35	Biochainin A	Safe	safe	Safe	safe
36	Donezepil	Safe	safe	Safe	safe

**Table 5:** Autodock binding energy scoring values of compounds on AChE and BACE-1

**Sl. No**	**Ligands**	**Proteins**	
		**AchE 4EY7 Affinity (Kcal.mol)**	**BACE-1 5HDZ Affinity (Kcal.mol)**
1	Petunidin	-7.19	-6.72
2	Peonidin	-7.69	-7.07
3	Pelargonidin	-7.42	-7.11
4	Malvidin	-8.69	-7.01
5	Delphinidin	-6.76	-6.2
6	Cyanidin	-8.01	-7.08
7	Tangeritin	-9.24	-6.76
8	Luteolin	-5.1	-6.3
9	Baicalein	-7.79	-6.96
10	Apigenin	-8.87	-6.38
11	Phloridzin	-7.63	-5.91
12	Phloretin	-7.9	-6.81
13	Chalcone	-8.06	-7.14
14	Chalconaringenin	-7.49	-6.3
15	Arbutin	-6.82	-6.38
16	Epicatechin gallate	-10.42	-7.47
17	Gallocatechin	-7.03	-7.9
18	Catechin	-7.97	-6.84
19	Taxifolin	-8.67	-6.58
20	Rutin	-8.59	-7.04
21	Quercitrin	-9.22	-7.6
22	Quercetin	-8.21	-6.54
23	Kaempferol	-8.08	-6.37
24	Isoharmnetin	-9.16	-8.96
25	Fisetin	-10.11	-9.81
26	Naringenin	-8.9	-6.55
27	Hesperidin	-8.51	-7.73
28	Hesperetin	-9.55	-6.42
29	Eriodictyol	-8.23	-6.29
30	Sterubin	-10.16	-8.96
31	Glycitein	-8.53	-6.55
32	Genistin	-9.92	-6.84
33	Formononetin	-8.38	-6.97
34	Daidzin	-8.93	-7.66
35	Biochainin A	-8.47	-9.81
36	Donepezil	-11.03	-8.83
